# Does the benefit of time for working memory arise at encoding or retrieval?

**DOI:** 10.3758/s13423-026-02926-3

**Published:** 2026-05-08

**Authors:** Eda Mızrak, Klaus Oberauer

**Affiliations:** 1 Department of Psychology, University of Zurich, Zürich, Switzerland Binzmuhlestr. 14, 8050; 2 Present Address: Centre for Brain and Behaviour, School of Biological and Behavioural Sciences, University of Queen Mary, London, UK Mile End Rd, Bethnal Green, E1 4DQ; 3 Department of Experimental Psycology, University of Oxford, Oxford, UK Life and Mind Building, S Parks Rd, OX1 3EL

**Keywords:** Working memory, Forward serial recall, Free time benefit, Random probed recall

## Abstract

Extended free time between the encoding of list items enhances immediate memory for serial order. Previous research suggests that this effect is predominantly proactive, free time improves memory for items presented after an extended interval but not for those presented before. This conclusion is based on two key findings. First, in forward serial recall, the benefit of free time increases with the serial position of the items (Mızrak & Oberauer, 2021, Oberauer, 2022b). Second, when a single inter-item interval is lengthened, the additional time primarily benefits items presented after that interval (Lu et al., 2024; Mızrak & Oberauer, 2021). Here we tested these two findings using both forward and random order recall and examined whether the relationship between free-time duration and the serial position of item differs when test order was changed. Our findings replicate the evidence for a proactive benefit of time in most conditions. Additional retroactive benefits of free time were apparent in some conditions. Retroactive benefits were more often observed when test order was random, in particular when participants could anticipate being tested in random order.

## Introduction

In immediate-serial recall tests, when items are presented at a slower pace, performance improves (Mızrak & Oberauer, 2021; Oberauer, 2022b; Souza & Oberauer, 2017; Tan & Ward, 2008).^1^ This beneficial effect has been attributed to the possibility of engaging in more maintenance mechanisms during the encoding process, where people covertly work on studied items by rehearsing, refreshing, or elaborating on them (Baddeley & Lewis, 1984; Bartsch & Oberauer, 2021; Camos et al., 2018; Tan & Ward, 2008). However, several findings call this interpretation into question.

**Fig. 1:**
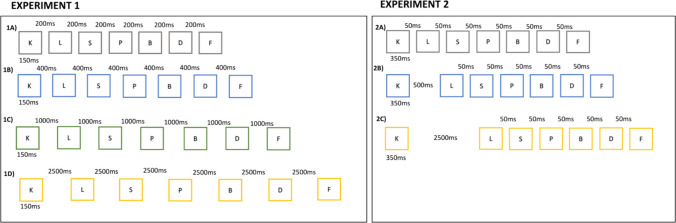
Example list presentation schedules in different conditions of Experiments 1 and 2. Each row shows the time course of list presentation in one condition. The numbers below each letter box show the presentation duration; the numbers above the letter boxes indicate the inter-stimulus interval (ISI; offset of preceding letter to onset of next letter), which was varied between conditions. Experiment 1 (left panel) shows the four levels of ISI. Experiment 2 (right panel): (**A**) No gap (baseline) condition; (**B**) short gap condition; (**C**) long gap condition. B and C show a gap in the first position, following the first list item. The gap appeared equally often in each of the six inter-item positions in the list

First, preventing articulatory rehearsal does not eliminate the time benefit (Baddeley et al., 1984; Longoni et al., 1993; Oberauer, 2022b). The time benefit therefore cannot be attributed to articulatory rehearsal. Second, the beneficial effect of longer inter-item time is predominantly proactive. This has been shown with two experimental designs. The *presentation-rate design* varies the list-wide presentation rate, so that all inter-item times in a list are shorter or longer (Fig. 1, left panel). The beneficial effect of a slower presentation rate interacts with serial position: It is absent for the first item, and increases over serial positions towards the last item, suggesting a cumulative effect of longer inter-item time for subsequently encoded items (Oberauer, 2022b). In the *single-gap design*, a single temporal gap of variable length is inserted between presentation of two items (Fig. 1, right panel). Compared to a shorter gap, a longer gap mostly improved memory of items that were presented after it, with little effect (Lu et al., 2024) or none at all (Mızrak & Oberauer, 2021) on items presented before. If time between items was used for maintenance processes, these processes would have to operate on items presented before but not after that time. Therefore, an experimental variation of inter-item time should be expected to have a mostly retroactive effect: It should affect items encoded before the manipulated time, rather than those encoded after it.

Besides maintenance, other processes could be responsible for the effect of inter-item time: (1) Free time between presentation of an item and the next could be used for short-term consolidation of the last-presented item, which means transforming a fragile memory representation into a more robust one (Jolicoeur & Dell'Acqua, 1998; Ricker et al., 2018). (2) Longer inter-item time might result in more change of a temporal context, resulting in larger temporal distinctiveness of items, which makes them easier to recall (Brown et al., 2007). (3) Free time could help replenish a limited cognitive resource that becomes partially depleted during encoding but replenishes over time (Popov & Reder, 2020; Reder et al., 2016). Simulations of these hypothetical processes (Oberauer, 2022b) have shown that they can explain the interaction of inter-item time with serial position in the presentation-rate design. Mızrak and Oberauer (2021) show that the resource-replenishment hypothesis can also explain the proactive benefit of longer gaps in the single-gap design, though it does not explain the retroactive benefit sometimes observed (Lu et al., 2024).

So far, the effects of inter-item time have been investigated only with tests of forward serial recall. This could be a problematic limitation because much of the evidence speaking to possible explanations of the effect comes from interactions of free time with serial position, either defined relative to the beginning of the list, or relative to the experimentally manipulated gap within the list. In forward serial recall, the serial position of an item in the presented list (input position) is confounded with its serial position in the recall sequence (output position). Therefore, whether input position or output position is responsible for the modulation of the free-time benefit remains ambiguous.

The explanations for the free-time benefit discussed so far share the assumption that longer free time improves memory as a function of input position independent of their output position. An alternative explanation is that longer free time results in representations that are more robust against interference from events between encoding and retrieval, including recall of other items (i.e., output interference; Cowan et al., 2002; Oberauer, 2003). Kowialiewski and Majerus (2024) have advanced this hypothesis. If it is true, then free time should be more beneficial for items later in the output sequence, because they suffer more interference from previous recall events. Consequently, in the presentation-rate paradigm (Fig. 1, left panel) free time should interact with output position rather than input position when the two are deconfounded. In the single-gap paradigm (Fig. 1, right) free time should have a benefit that increases with output position regardless of gap location. The aim of the present work is to deconfound input and output position by testing immediate memory for lists with probed recall, probing items in a random order that is uncorrelated with presentation order. Experiments 1a, 1b, and 1c use the presentation-rate design, and Experiments 2a and 2b use the single-gap design.

## General method

### Participants

Participants were healthy young adults, 18–35 years old; *N* = 48, 201, and 237 for Experiments 1a, 1b, and 1c, respectively, and *N* = 97 and 175 in Experiments 2a and 2b, respectively. Sample sizes were chosen based on previous experiments that have shown beneficial effects of longer inter-item intervals; we approximately doubled those numbers to compensate for the less controlled environment of online experiments. Experiments lasted between 30 and 60 min. Participants were recruited from the online data collection platform Prolific and were reimbursed with 9 GBP per hour for their time. In all experiments, participants completed an online informed consent form and were debriefed at the end. The experimental protocol was in accordance with the ethics guidelines of the Faculty of Arts and Social Sciences of the University of Zurich.

We excluded participants based on the following criteria: They performed very poorly, that is, below 0.1 on average for multiple conditions (i.e., more than three conditions). Such low performance indicated that participants did not do the task properly. With this exclusion criteria, we excluded eight participants for Experiment 2a and eight participants for Experiment 2b.

### Materials

For each list in Experiments 1a, 1b, 2a, and 2b, seven consonants were randomly drawn without replacement from the consonants of the English alphabet. In Experiment 1c, lists consisted of six English nouns that were drawn at random from a large pool which were from Devereux et al. (2014). We selected words consisting of between four to eight letters and there were 474 words overall.

### Procedure

#### Experiment 1a

The duration of inter-stimulus intervals (ISIs) was manipulated between lists. Each trial started with a fixation cross in the screen center for 500 ms. Then the seven consonants were presented one by one in the screen center. Each consonant was shown for 150 ms; then the screen went blank for the duration of the inter-stimulus interval (ISI), which could be 50 ms, 250 ms, 850 ms, or 2,350 ms. ISI durations were the same for all inter-item positions in a list. After the last ISI, the word “Forward” or “Random” was shown for 1 s to inform about the recall order. Offset of that message was immediately followed by a centrally presented red digit that indicated the list position to be recalled next. In the forward-recall condition the digits appeared in the order 1 to 7, whereas in the random-recall condition they appeared in a new random order in every trial. In response to each digit the participant had to type a letter, which was displayed on the screen for 500 ms, replacing the digit. After a 500—ms blank screen the next digit was presented. After the last letter had been typed, there was a 1.5—s inter-trial interval, after which the next trial started automatically.

The experiment was organized into blocks of eight trials. Within each block, each combination of the four ISIs with the two recall orders was realized in one trial. The eight trials were presented in a random order. In this way, participants could not know which recall order they would be tested in before list presentation had been completed. Participants worked through eight test blocks. They were preceded by one practice block (also consisting of eight trials).

#### Experiments 1b and 1c

These experiments used the same method as Experiment 1a with small variations that moved them closer to the Experiment 3 of Kowialiewski and Majerus (2024), which used a similar design. Their experiment realized only the random recall order, and therefore participants could anticipate that they would be tested in random order. To replicate that condition, Experiment 1b was identical to 1a but varied recall order between subjects (*N* = 107 in the forward-recall and *N* = 94 in the random-recall condition). Experiment 1c was identical to Experiment 1b except that lists were composed of six nouns, as in Kowialiewski and Majerus (2024) (*N* = 106 in the forward-recall and *N* = 131 in the random-recall condition). Each word was presented for 450 ms, and each consonant was presented for 150 ms which was followed by a fixed ISI. There were eight trials per ISI condition for both consonants and words. ISI conditions for consonants were 50, 250, 850, and 2,350 ms and for words were 50, 550, 1,550, and 3,550 ms.

#### Experiments 2a and 2b

There were three main conditions. In the baseline condition there was no gap; all ISIs were 50 ms. In the gap conditions there was a deviant ISI at one inter-item position, which was either short (ISI = 500 ms) or long (ISI = 2,500 ms). Both created a temporal gap against the background of the remaining standard ISIs, which were all 50 ms. Such a gap is known to give rise to temporal grouping (Ryan, 1969a), but as Ryan (1969b) has shown equivalent grouping effects for short and long gaps, we expected no difference in grouping effects between the short-gap and the long-gap condition. We investigated whether, on top of the common grouping benefit, the extra free time given in the long-gap condition improves the memory for items preceding the free time or items subsequent to free time. There were six positions in the study list where the gap could be inserted: following the first item (1 +), second item (2 +), third item (3 +), fourth item (4 +), fifth item (5 +), or sixth item (6 +). In Experiment 2a, there were 13 conditions: The baseline condition without a gap, and two gap durations, each with six gap positions with three trials per condition and 39 trials in total. In Experiment 2b, there were the same 13 conditions for each recall type (i.e., forward serial vs. random cued) with five trials per condition and 130 trials in total.

Each trial began with a central fixation point presented for 500 ms followed by the study list presentation. Each list item was presented for 300 ms followed by a blank screen for the duration of the ISI. The last ISI was followed by a delay of 1,000 ms before testing commenced. In Experiment 2a, items were always prompted in random order. Recall of each item was prompted by a digit indicating the list position of the item to be recalled next. In Experiment 2b, the recall order was cued after list presentation by a digit on the screen. If recall was in random order, it was prompted as in Experiment 2a. If recall was in forward order, participants were asked to type in the letter sequence in their order of presentation. The recall type was cued by the colour of the input box on the screen. For random recall, participants saw a red input box with a red digit presented next to it. For forward recall, participants saw a black input box with a black digit presented next to it. In the case of forward recall, the digits appeared in the order 1 to 7, and for random recall, the digits appeared in a random order for each trial. There was also a small instruction at the bottom of the screen which was “type in the letter for this position” for random recall and “type in the letters in the order you have seen them” for forward recall. Once they typed in one letter, the screen went blank for 100 ms and they were asked to type in the next one.

### Data analysis

We used Bayesian Generalized Linear Models with a logistic link function to predict the number of correctly recalled items in each design cell for each participant. An item was counted as correct if it was recalled in its correct list position. The models were run with the *brms* package (Bürkner, 2025) in R (R_Core_Team, 2024) with eight chains of 30,000 iterations each. We contrast-coded binary predictor variables (i.e., output order) and z-standardized continuous predictor variables (i.e., ISI, serial position, lag), so that effect sizes are standardized. We used Cauchy priors with scale = 0.5 for the effect sizes of fixed effects, and Gamma (1, 0.01) priors for random effects.

For Experiments 1a, 1b, and 1c we used model comparisons through the Bayes factor (BF) estimated through the bridge-sampling algorithm (Gronau et al., 2017). We fit the full model, including random intercept and all random slopes, to each data set. We first assessed the evidence for the inclusion of random slopes by comparing the full model as reference model to a model removing the random slopes. Random slopes were kept in the reference model if this model comparison supported them (Oberauer, 2022a). We next evaluated fixed effects one by one, starting with the highest-level interaction, by comparing the current reference model with a model removing the fixed effect in question (while keeping the corresponding random effect). If the BF yielded evidence more against than in favour of the fixed effect (i.e., BF < 1), the fixed effect was removed from the reference model for subsequent model comparisons. We report BFs in favour of an effect as BF_10_, and BFs in favour of the null hypothesis as BF_01_. For Experiments 2a and 2b we used a similar approach: We fit the full model and used it to estimate BFs for individual fixed effects through the Savage-Dickey density ratio (Wagenmakers et al., 2010).

### Results

#### Experiment 1a

We ran a first analysis of all data including fixed effects of input position, ISI, and output order and all their interactions, as well as a random intercept and random effects of all main effects and two-way interactions. The model equation in Wilkinson format is:

$${N}_{correct}\left|{N}_{responses}\sim inpos*ISI*outorder+\left(1+inpos*ISI+inpos*outorder+ISI*outorder\right)\right|id$$

A second analysis on the trials with random output order used ISI and output position and their interaction as fixed effects, together with a random intercept and random slopes of both main effects:

$${N}_{correct}\left|{N}_{responses}\sim outpos*ISI+\left(1+outpos+ISI\right)\right|id$$

**Fig. 2:**
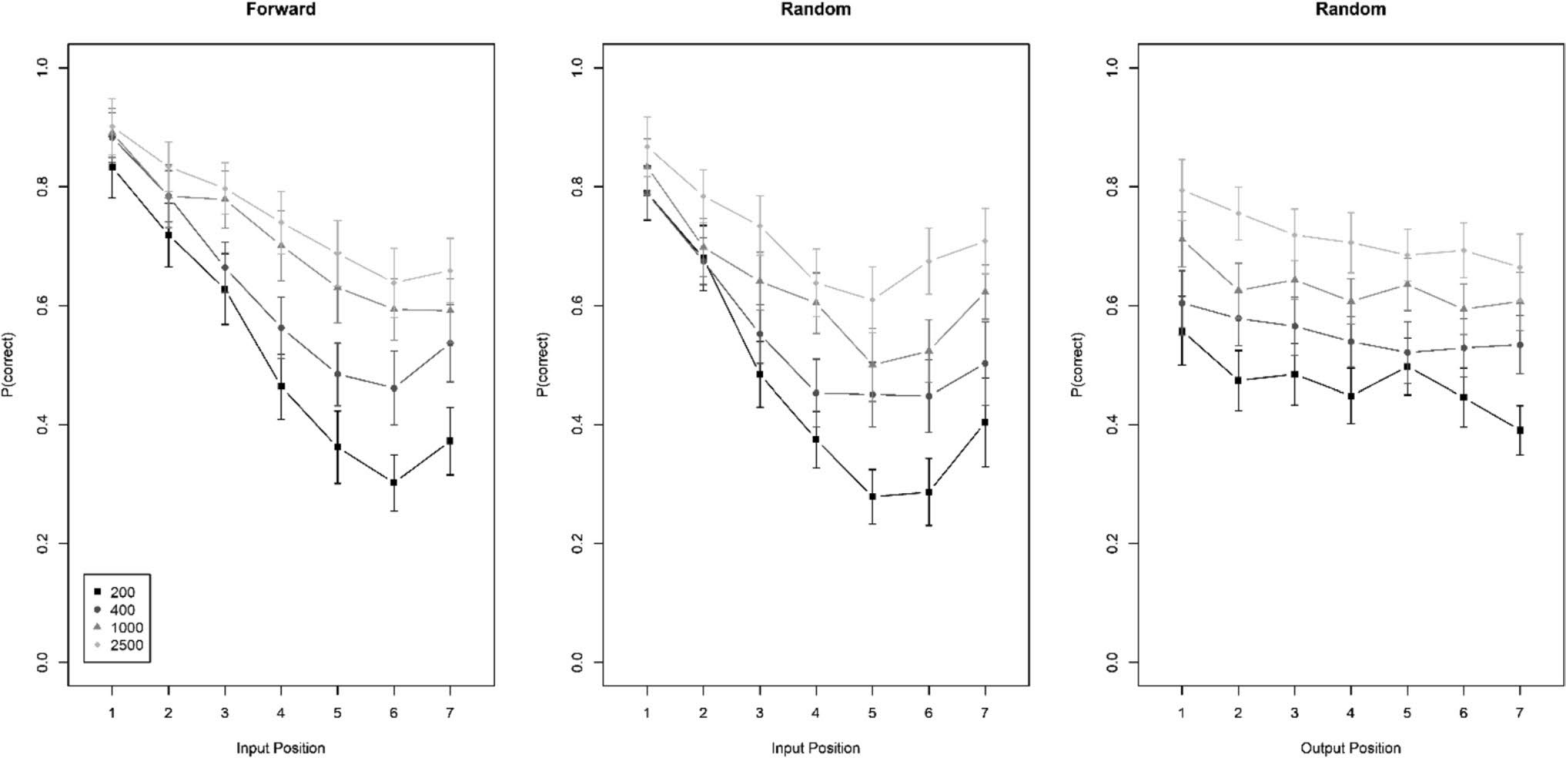
Accuracy over input serial position for forward and random recall order, and over output position with random recall order, Experiment 1a. Error bars are 95% confidence intervals for within-subject comparisons

The first two panels of Fig. 2 show serial-position curves for each ISI condition over input position. With forward recall order, the pattern replicates the interaction of input position with presentation rate (Oberauer, 2022b): Longer ISIs resulted in better performance, and that benefit increased with input position. With random recall order, we observed the same interaction. Across both recall orders, that interaction received unambiguous support (BF_10_ = 602). There was evidence against the three-way interaction of input position, presentation rate (ISI), and recall order (BF_10_ = 0.054; BF_01_ = 18.5), implying that recall order did not change the degree to which the free-time benefit increased over input positions.

The third panel of Fig. 2 shows serial-position curves over output position in the random output order. For this condition we analyzed whether ISI interacted with output order; it did not (BF_01_ = 15.3).

Kowialiewski and Majerus (2024) published an experiment varying presentation rate consistently throughout the list and testing in random order. As opposed to our Experiment 1a, their Experiment 3 showed a free-time benefit that was largely additive with input position, which implies that the effect is not purely proactive. They also found a tendency for the free-time benefit to increase with output position. There are a few differences in the details between our experiment and theirs. We identified two differences that could plausibly be responsible for the different outcomes. First, they did not include a forward-recall condition. Therefore, participants knew that they would be tested in a random order in every trial. A person's expectation of test order could reasonably influence how they use free time to encode a list, thereby affecting how the benefit of slower presentation rates is distributed across serial positions. Second, they used concrete words rather than letters as items. We ran two follow-up experiments to clarify whether these variables could explain the difference between their findings and ours. Experiments 1b and 1c varied recall order between subjects, so that participants in the random-order group could anticipate being tested in random order. Whereas Experiment 1b still used letter lists, in Experiment 1c we tested memory for word lists.

#### Experiment 1b

**Fig. 3:**
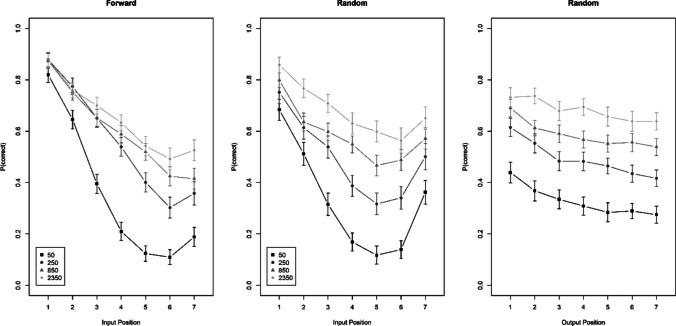
Accuracy over input serial position for forward and random recall order, and over output position with random recall order, Experiment 1b

Figure 3 shows the results in the same format as for Experiment 1a. The benefit of free time again increased with input position in both recall-order conditions. The interaction received unambiguous support (BF_10_ = 9.1 × 10^9^). Different from Experiment 1a, here the three-way interaction was supported, if only weakly (BF_10_ = 4.9). With random recall order the interaction of presentation rate with input position was less pronounced, and the evidence for it was ambiguous (BF_10_ = 0.77). In the group with random recall order, presentation time did not interact with output order (BF_01_ = 33).

#### Experiment 1c

**Fig. 4:**
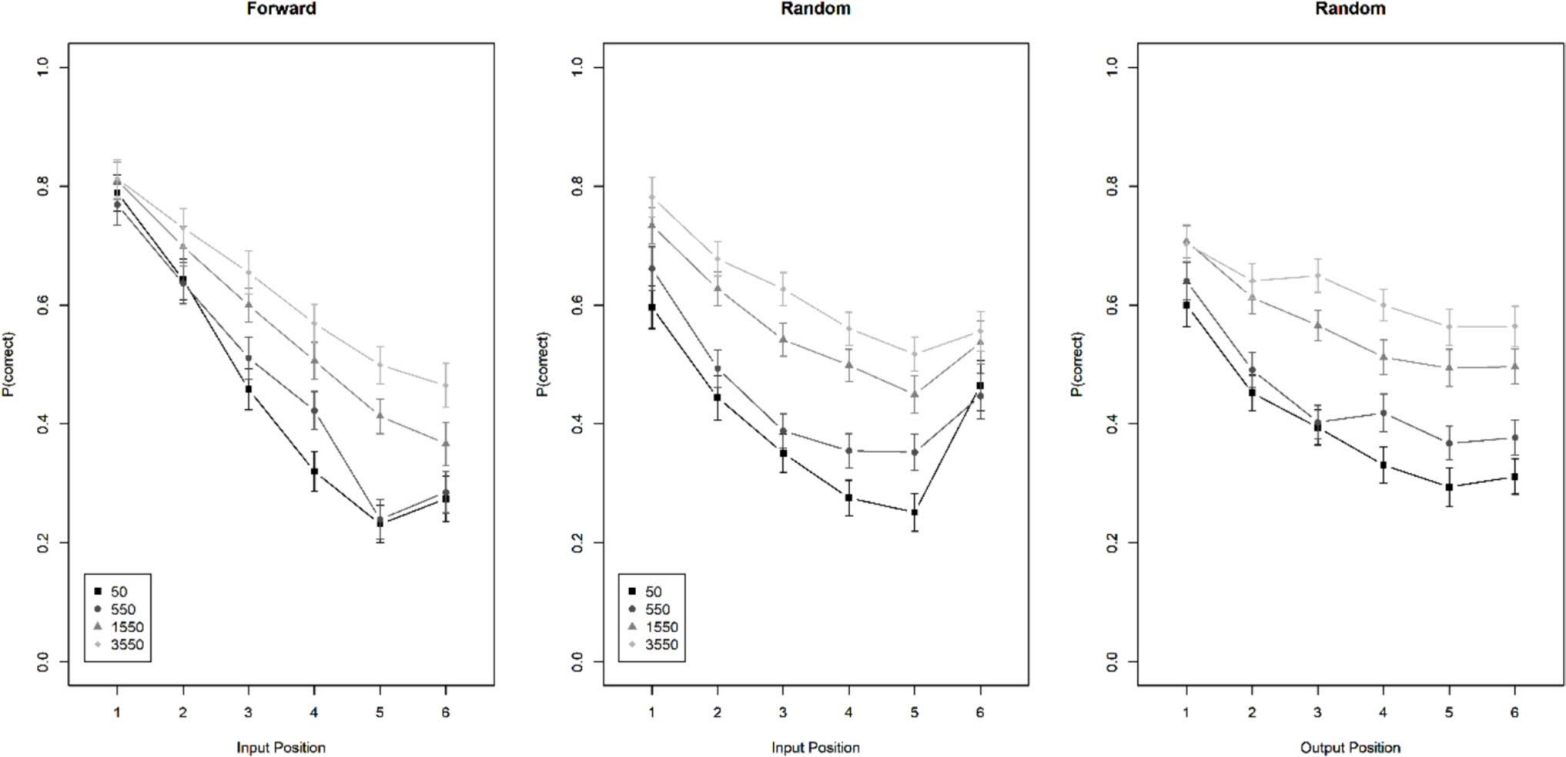
Accuracy over input serial position for forward and random recall order, and over output position with random recall order, Experiment 1c

Figure 4 presents the results. This experiment, using word lists in a between-subjects manipulation of recall order, generated a result pattern similar to that of Kowialiewski and Majerus (2024, Exp. 3). Across both recall orders, the interaction of presentation time and input position was again strongly supported (BF_10_ = 4.0 × 10^7^), but it was much less pronounced with random than with forward recall order, as reflected in clear evidence for the three-way interaction (BF_10_ = 6.9 × 10^7^). With random recall order, there was no evidence for the interaction of free time with input position (BF_10_ = 0.33). Moreover, for the first time we found that the benefit of free time increased over output position in the random recall-order group (BF_10_ = 4.6 × 10^5^).

#### Experiment 2a

**Fig. 5:**
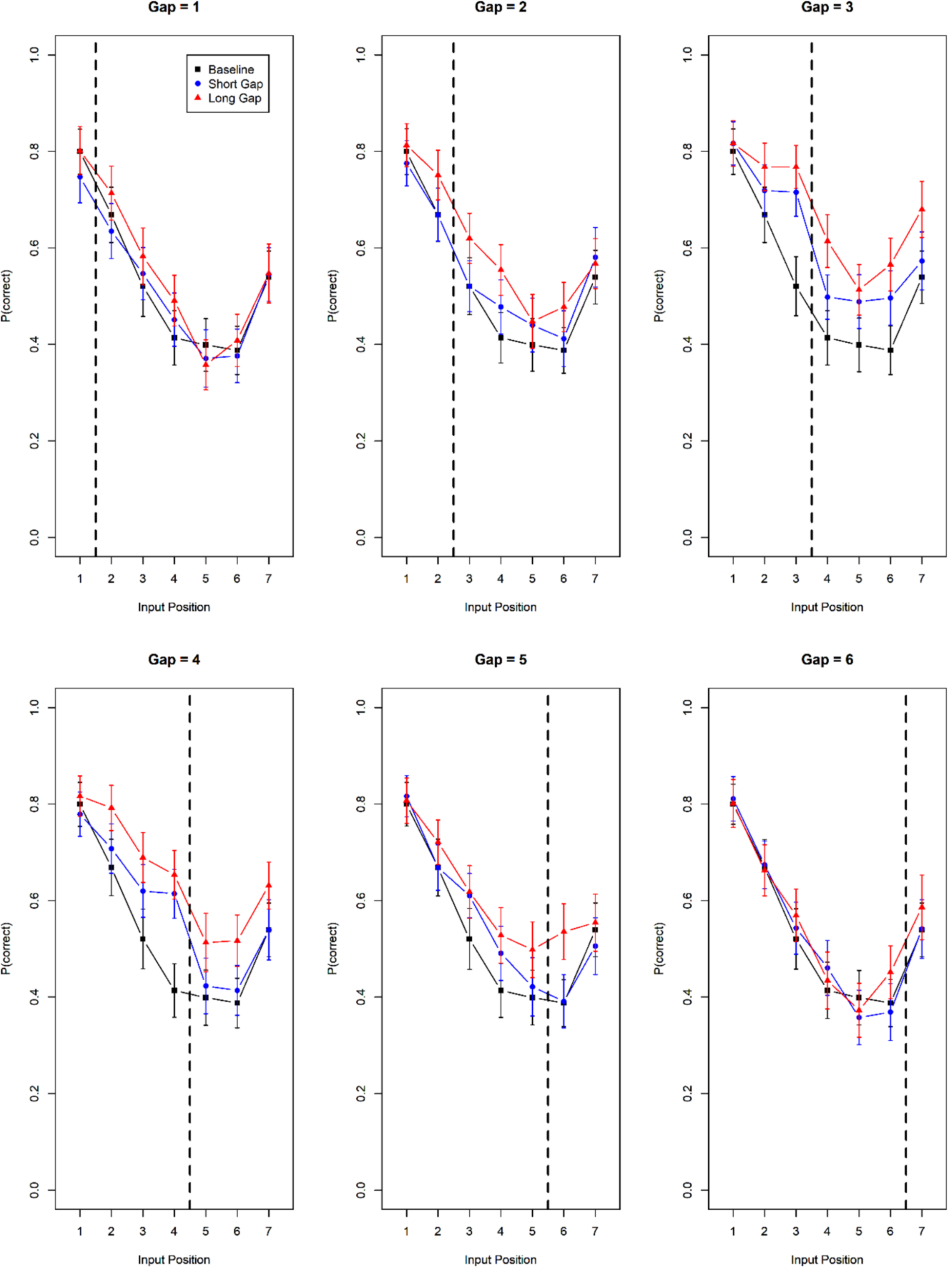
Serial-position effects by gap position, Experiment 2a. The dashed vertical bar indicates the position of the gap in the short-gap and long-gap conditions. Error bars are 95% confidence intervals for within-subjects comparisons

Figure 5 presents the serial-position curves over input position for the three main conditions (baseline, short gap, and long gap). Each panel shows data for one gap position; the baseline data are repeated across panels. The short gap led to better performance than the baseline, and the long gap improved performance over and above the short gap. When split by gap position, these effects are overlaid with substantial measurement noise. A clearer picture emerges when we combine the data across all gap positions. In Fig. 5 accuracy in the three main conditions is shown as a function of each item’s lag relative to the gap. The lag is the item’s input position minus the gap position. Hence, negative lags indicate items preceding the gap, and positive lags indicate items following the gap. For instance, when the gap position is between items 3 and 4, input position 1 receives lag −3; input position 3 receives lag −1; input position 5 receives lag + 2.

**Fig. 6:**
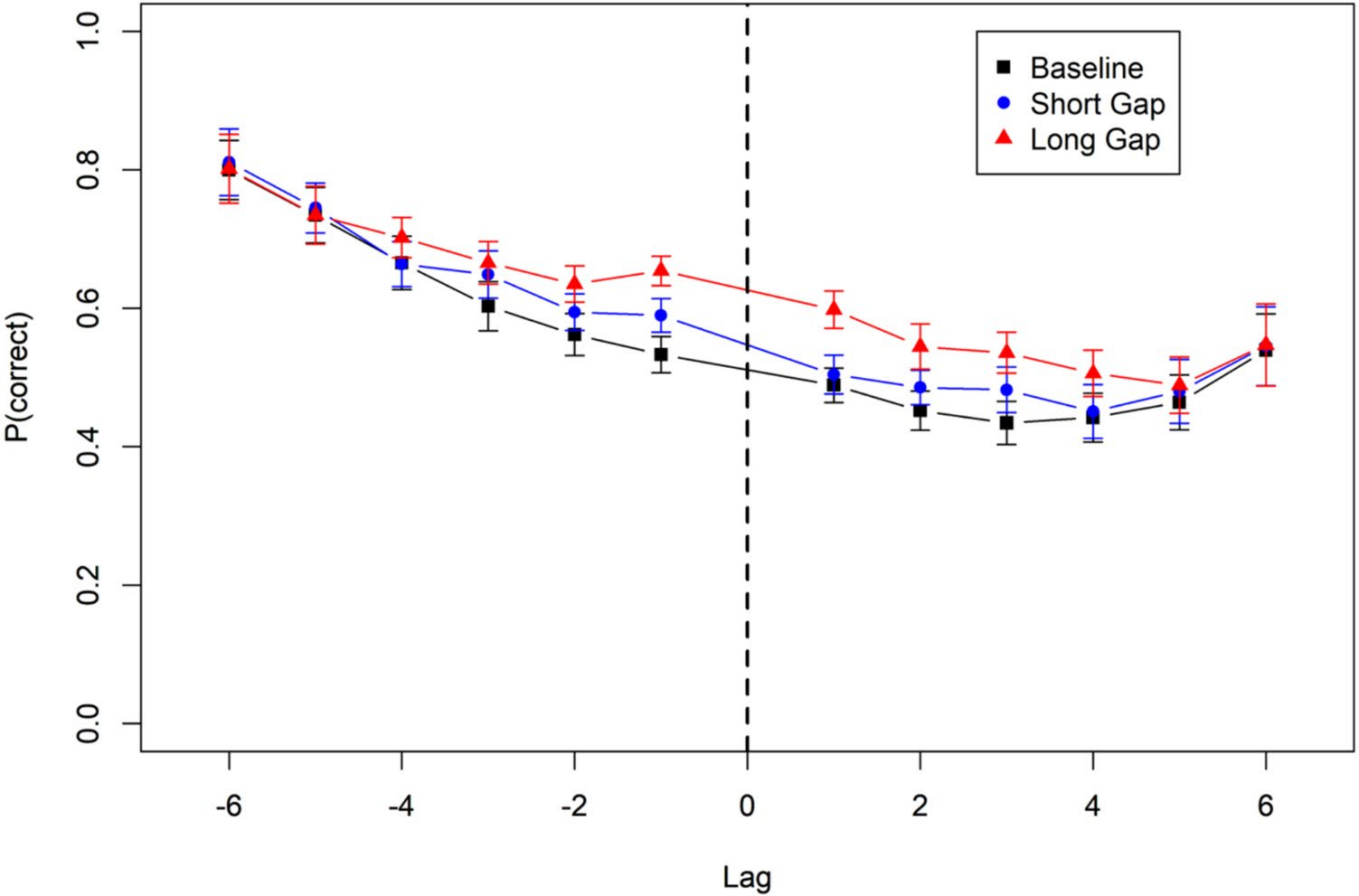
Accuracy by lag from the gap, Experiment 2a. The vertical broken line denotes the location of the gap for which the duration was manipulated. Data from the baseline condition were assigned to a lag level by replicating them six times, each time with another virtual gap location. Lag was computed relative to that virtual gap location in the same way as it was computed relative to the actual gap location in the Gap conditions. Error bars are 95% confidence intervals for within-subject comparisons

Figure 6 shows that the beneficial effect of a longer gap extended into the retroactive direction (i.e., negative lags) as well as the proactive direction (i.e., positive lags). This was the case both for the contrast between baseline and short gap, and for the contrast between short and long gap. For the statistical analysis we broke down the lag variable into two orthogonal predictors. The sign of the lag reflects the direction of the effect (retroactive vs. proactive), whereas the absolute value of the lag reflects whether the effect is local, confined to small lags, or global, extending to larger lags. The three main conditions were coded by two contrasts. The first (C1) compares baseline to short lags and is intended to reflect grouping effects. The second (C2) compares short and long lags and is intended to capture the effect of longer versus shorter free time. The full model is:

$${N}_{correct}\left|{N}_{responses}\sim abs\left(lag\right)*sign\left(lag\right)*C1+abs\left(lag\right)*sign\left(lag\right)*C2+\left(1+abs\left(lag\right)*C1+abs\left(lag\right)*C2+sign\left(lag\right)*C1+sign\left(lag\right)*C2\right.\right|id$$

Table 1 shows the BFs for the contrast between baseline and short-gap condition, and the contrast between the short-gap and long-gap conditions, as well as their interactions with the sign and the absolute value of the lag. The contrast of long versus short gaps (C2) measures the free-time effect independent of grouping effects. This contrast did not credibly interact with lag sign, reflecting an equally proactive and retroactive beneficial effect of free time. The interaction with absolute lag shows that the effect diminished with longer lags.

**Table 1:** Bayes factors in favour of effects in Experiments 2a and 2b

Effect	Experiment 2a	Experiment 2b (Forward)	Experiment 2b (Random)	Experiment 2b (Interaction with Test Order)
Contrast C1 (Base-Short)	0.49	0.05	2.5	0.23
Contrast C2 (Short-Long)	64	0.10	550,149	0.92
C1(Base-Short) x Sign(Lag)	0.11	0.05	0.02	0.02
C2(Short-Long) x Sign(Lag)	0.16	9.67	0.03	6.4
C1(Base-Short) x abs(Lag)	142	1.7e13	423	4.3
C2(Short-Long) x abs(Lag)	17,878	3.6e20	4.8	4048
C1(Base-Short) x abs(Lag) x Sign(Lag)	0.13	273	0.34	0.04
C2(Short-Long) x abs(Lag) x Sign(Lag)	0.04	20	0.02	0.32

A second analysis investigated whether the free-time benefit from a longer gap increases with output position. The model averaged over all gap positions:

$${N}_{correct}\left|{N}_{responses}\sim outpos*C1+outpos*C2+\left(1+outpos+C1+C2\right)\right|id$$

**Fig. 7:**
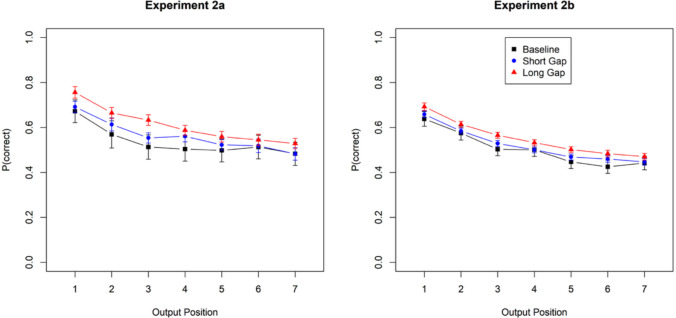
Accuracy by output position for random-order recall, Experiment 2a (**left**) and Experiment 2b (**right**). Error bars are 95% confidence intervals for within-subject comparisons

Figure 7 (left panel) shows that performance declines with output position, reflecting the commonly observed output interference. The beneficial effect of a longer over a shorter gap was largely additive with output position, with a weak tendency to decrease. The BF_10_ for the interaction between output position and the Short-Long contrast (C2) was 3.0. These results reject the hypothesis that free time protects items from adverse effects of recalling other items

#### Experiment 2b

**Fig. 8:**
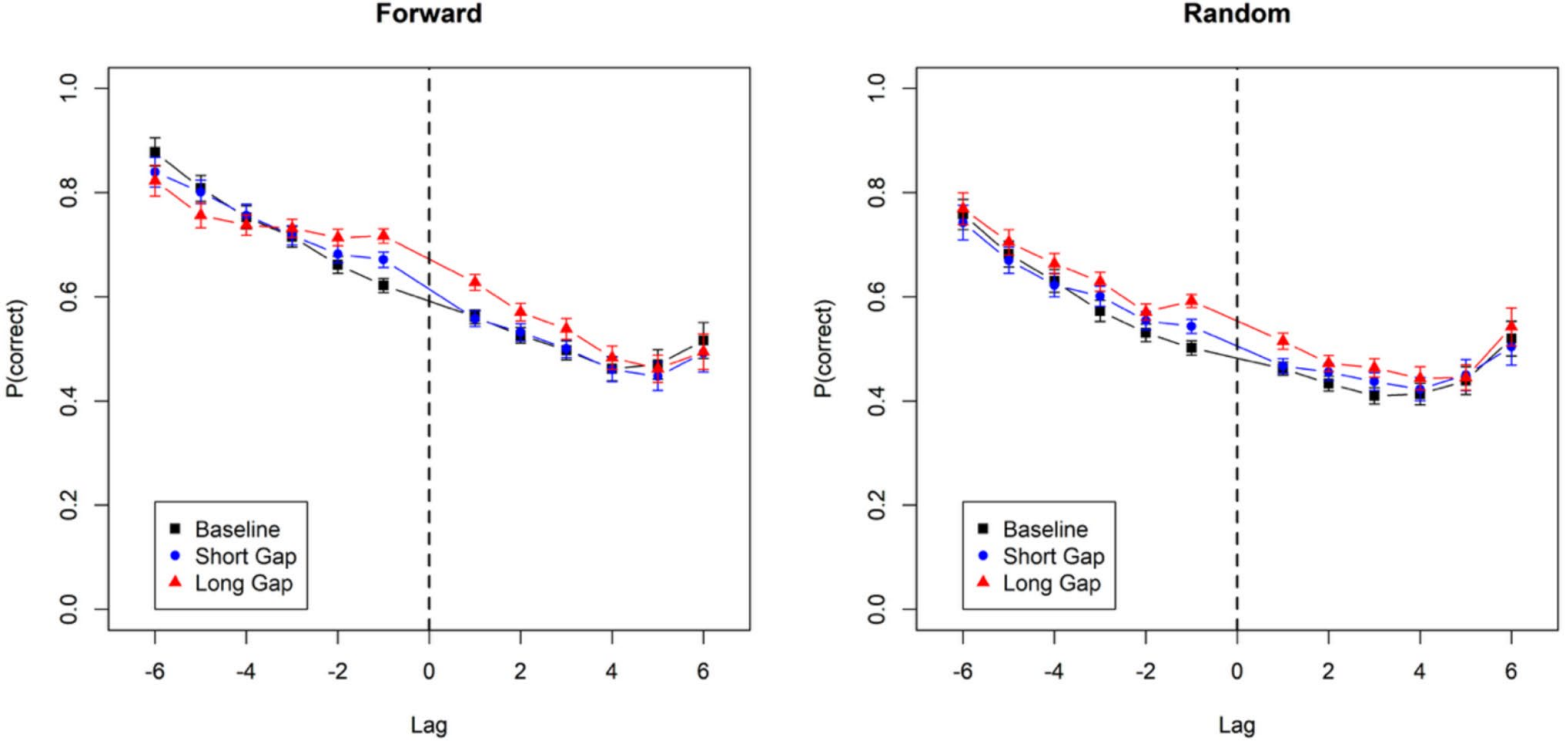
Accuracy by lag from the gap, Experiment 2b. The vertical broken line denotes the location of the gap for which the duration was manipulated. Error bars are 95% confidence intervals for within-subject comparisons

Figure 8 shows accuracy as a function of gap condition and lag for forward and random recall orders. The data for the forward recall direction show a pattern similar to the one in Mizrak and Oberauer (2021), with an extended proactive benefit of longer compared to shorter gaps. In addition, there was a retroactive benefit that was limited to short lags and reversed at longer lags. This retroactive benefit was more pronounced in the present data than in Mizrak and Oberauer (2021). With random recall order, there was again an extended proactive benefit of longer free time, accompanied by a retroactive benefit that extended further into larger (negative) lags than with forward recall.

Table 1 summarizes the BFs for analyses run separately for forward and random recall direction, as well as the BFs for the interaction of all effects with output order (forward vs. random).^2^ Across both Experiment 2a and Experiment 2b these analyses revealed strong evidence for an interaction of gap duration with absolute lag, reflecting the finding that the free-time benefit was larger closer to the gap. In the forward-recall condition of Experiment 2b, gap duration interacted with lag sign, providing evidence for more proactive than retroactive benefits of longer gaps. This confirms the results of Mızrak and Oberauer (2021) and Lu et al. (2024), who found more proactive than retroactive beneficial effects of free time. By contrast, in the random-recall conditions of Experiments 2a and 2b, there was evidence against an interaction of gap duration with lag sign, implying that the free-time benefit was about equally strong in proactive as in retroactive direction.

The right panel of Fig. 7 shows the data from the random recall order over output position. Again, the beneficial effect of free time was additive with output position, speaking against the hypothesis that free time protects items against output interference. The BF_10_ for the interaction of output position and the Short-Long contrast was 0.023.

## Discussion

Longer free time in between encoding of list items is beneficial for immediate memory of serial order. Previous work has found that this beneficial effect of free time is primarily proactive: Free time improves memory for items presented after the free-time interval but not items presented before. This conclusion rested on two observations. First, in forward serial recall, the free-time benefit increased with serial position (Mızrak & Oberauer, 2021; Oberauer, 2022b). Second, when a single inter-item interval is increased, that added time primarily benefits items presented after that interval (Lu et al., 2024; Mızrak & Oberauer, 2021). Here we investigated whether these interactions of free-time duration with the serial position of item presentation changed when lists are tested in random rather than in forward order. We found that they do, though not consistently – additional variables play a role, too.

In Experiment 1a, free time was varied for all inter-item intervals of a list. The beneficial effect of free time increased with input serial position regardless of whether lists were tested in forward or in random order. In Experiments 1b and 1c, in which participants could anticipate the test order (forward or random), this interaction was no longer observed; free time was about equally beneficial at all input positions. This implies that free time also has a retroactive benefit, which could arise from participants using free time for rehearsing, refreshing, or elaborating previously presented items. Experiments 1a through c suggest that participants are more likely to do this when they anticipate being tested in random order than when they cannot anticipate this test order.

In Experiments 2a and 2b, the free time added in a single gap had an extended proactive beneficial effect regardless of recall order. A new observation in these experiments is the additional retroactive benefit of a single prolonged gap. Such a retroactive benefit has been observed before (Lu et al., 2024) but only with other conditions (faster presentation rate) or materials (words) than used here. We found that the retroactive effect was affected by recall order: It extended more into longer lags with random recall order.

Hence, both sets of experiments show that a retroactive benefit of free time becomes more likely when recall order is random. Experiments 1a through 1c suggest that this happens only when participants know ahead of time that test order will be random, suggesting that participants use free time differently when they anticipate being tested in random order. However, in Experiment 2b they could not anticipate this, casting doubt on a role of different encoding processes for whether or not free time has a retroactive effect. The observed pattern could be explained by the hypothesis of Kowialiewski and Majerus that free time protects items against output interference. This would lead to an increase of the free-time benefit with output position. In the forward-recall condition later output positions are also later input positions, and this could explain why the free-time benefit increases with input position in the presentation-time design, and is most pronounced after the gap, that is, later in the list, in the single-gap design. In the random-recall condition input and output position are uncorrelated, and hence, a free-time benefit that increases with output position would not interact with input position.

This prediction matches with the findings from Experiment 1c, and from Experiment 3 of Kowialiewski and Majerus (2024). However, this explanation appears to work only for experiments with word lists. In the random recall order of Experiments 1a and 1b, the effect of presentation rate did not increase with output position. Moreover, in Experiments 2a and 2b the beneficial effect of a longer gap did not increase with output position, speaking against the hypothesis that free time generally protects against output interference.

**Fig. 9:**
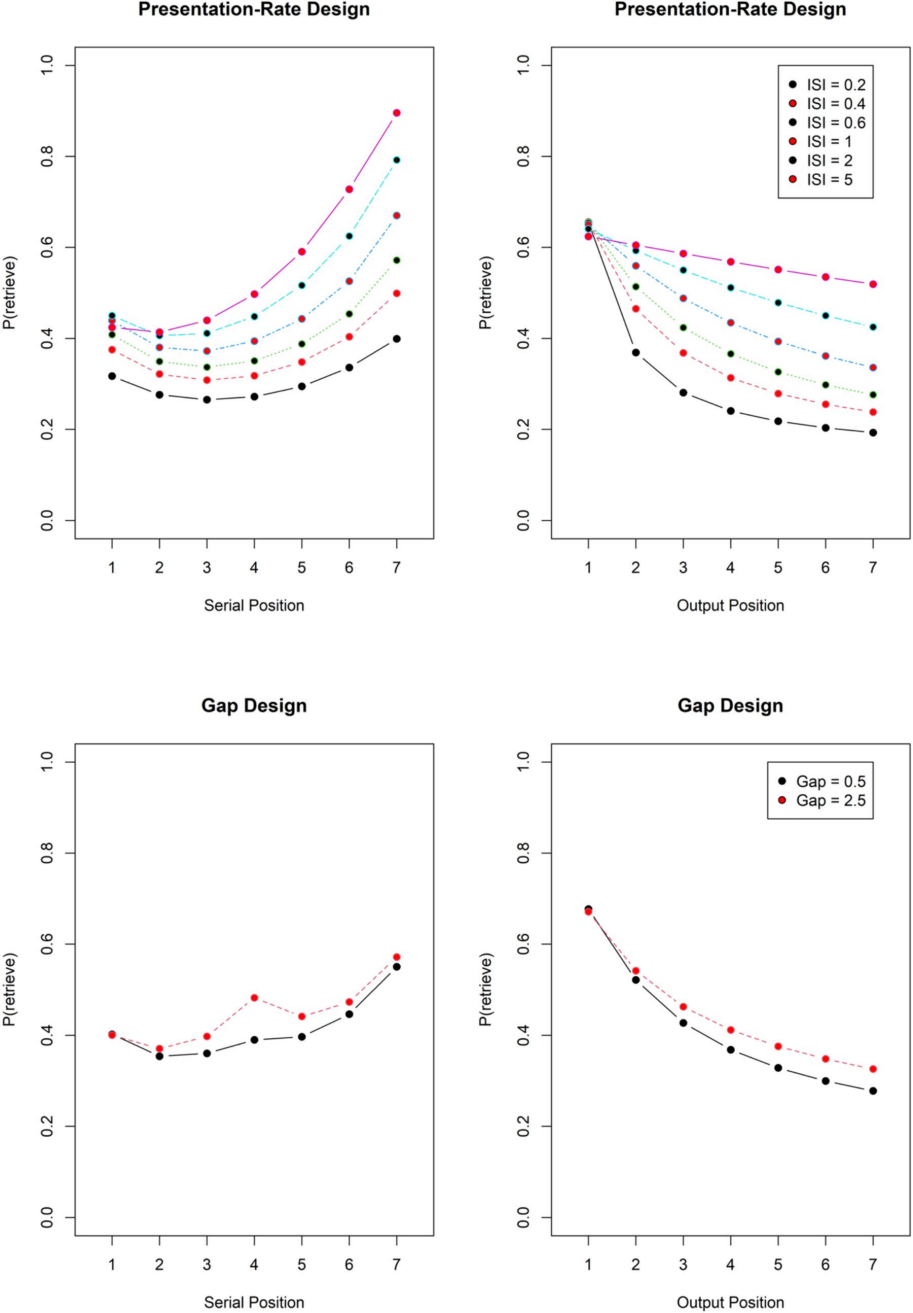
Simulations of temporal-distinctiveness predictions with the SIMPLE model. The simulation of the single-gap design placed the gap between the third and the fourth item, as indicated by the vertical black line. The generalization parameter in SIMPLE was set to c = 5 because that value gave a good approximation to the observed overall performance

Another explanation of the free-time effect that entails an interaction of input and output position is temporal distinctiveness. Longer free time separating items increases their distinctiveness; longer delays between encoding and retrieval reduce distinctiveness (Brown et al., 2007). As the output order affects the delay between encoding and retrieval of items in different input positions, temporal distinctiveness could explain why output order affects how the free-time benefit interacts with input position. To evaluate whether temporal distinctiveness could explain our findings, we simulated accuracy as a function of input and output position with the SIMPLE model of temporal distinctiveness (Brown et al., 2007) for both experimental designs. Figure 9 shows the results. Temporal distinctiveness predicts that the free-time benefit increases with output position in both designs, which matches our findings in Experiment 1c, but is contrary to the results of the other four experiments. Therefore, it does not provide a viable explanation for our results.

In sum, the present experiments, together with previous results from the presentation-rate and the single-gap paradigm, show a consistent proactive benefit of free time between encoding of list items. In addition, there is evidence for a retroactive benefit of free time, which appears to be more volatile: It is found in some experiments and experimental conditions but not in others. The same is true for the increase of free-time benefits with output position, which was observed only when word lists were tested.

These observations could be explained by the combination of a basic mechanism of memory that consistently generates the proactive benefit, together with the effects of encoding processes that sometimes do, sometimes do not use available free time, and cause a retroactive benefit when they do. The basic mechanism responsible for the proactive benefit could be a resource that is partially depleted by encoding an item and gradually replenished during free time (Mızrak & Oberauer, 2021; Popov & Reder, 2020). The retroactive benefit could arise from processes such as short-term consolidation that benefits from extended free time when the baseline presentation time of an item is not sufficient to complete consolidation (Lu et al., 2024), or elaboration that benefits from extended free time when people choose to engage in it.

## Data Availability

The anonymized data are available on the Open Science Framework (OSF) at: https://osf.io/b9zqv/

## Footnotes

1 This is the case regardless of whether or not items are presented for a longer time in the slower-paced conditions (Oberauer, 2022b).

2 The statistical model for the joint analysis was the same as for Experiment 2a with the addition that output order was included as a further predictor in all interaction terms for both fixed and random effects.

## Code availability

All materials including scripts for programming the experiments and scripts for data analysis are available at: https://osf.io/b9zqv/

## Declarations

### Open practices statement

All materials including scripts for programming the experiments and scripts for data analysis and the anonymized data are available on the OSF at: https://osf.io/b9zqv/. The experiments were programmed using the free and open online experiment builder lab.js (Henninger et al., 2024). Data analyses and plotting were conducted using open-source R and the R-packages.
